# Supplementary material for: PCK1 as a potential hub gene in distinguishing lactate metabolism between rheumatoid arthritis and osteoarthritis
Source: PeerJ. 2025 Jul 31;13:e19661. doi: 10.7717/peerj.19661 (PMC12318502; doi:10.7717/peerj.19661)
Supplement: Supplemental Information 1 [file peerj-13-19661-s001.docx]

Supplementary Material

# Supplementary table 1. The target genes and their primer sets

| Gene | Forward Primer | Reverse Primer |
| --- | --- | --- |
| PCK1 | GGAAGCCTGGACAGCCTACC | TCCTCAGAGCCGTCACAGATG |
| GAPDH | CATGTTCGTCATGGGGTGAACCA | AGTGATGGCATGGACTGTGGTCAT |


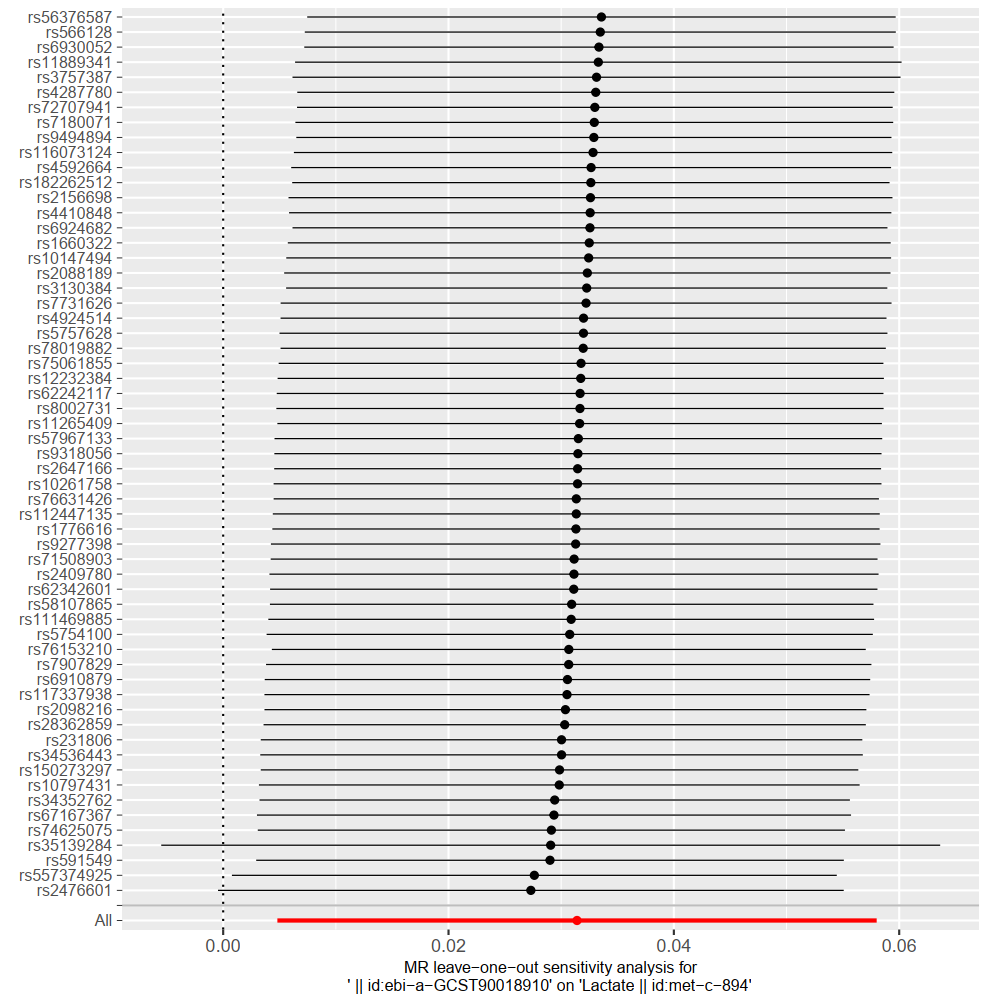
**Supplementary Fig.S1.** **Leave-one-out analysis of RA**


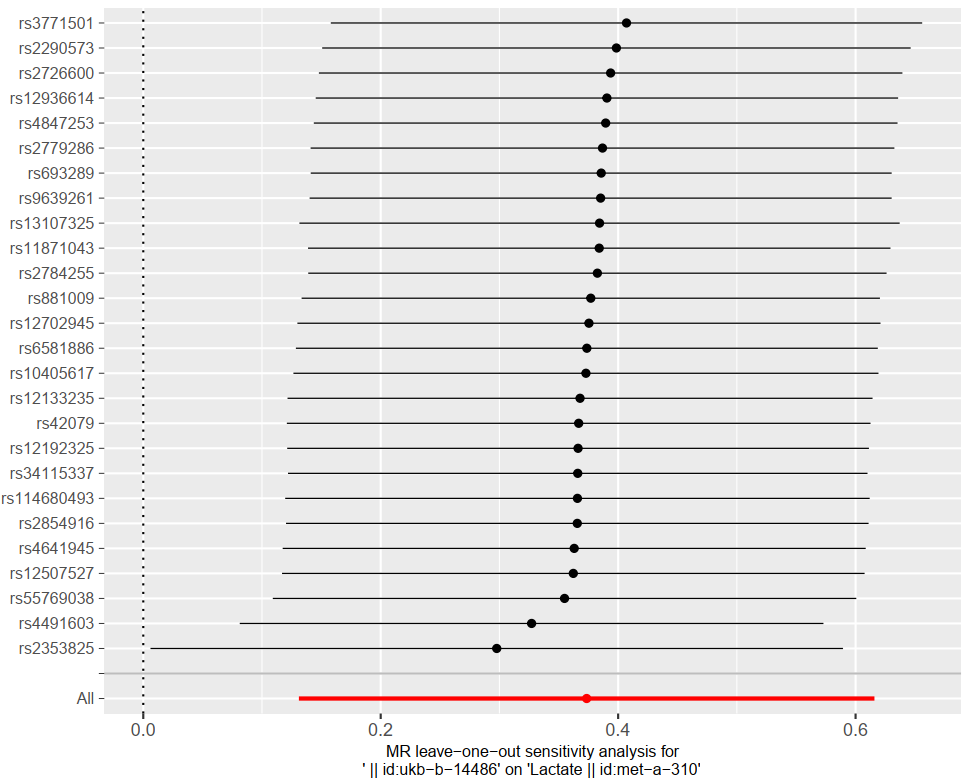
**Supplementary Fig.S2.** **Leave-one-out analysis of OA**
