# Supplementary material for: PCK1 as a potential hub gene in distinguishing lactate metabolism between rheumatoid arthritis and osteoarthritis
Source: PeerJ. 2025 Jul 31;13:e19661. doi: 10.7717/peerj.19661 (PMC12318502; doi:10.7717/peerj.19661)
Supplement: Supplemental Information 4 [file peerj-13-19661-s004.docx]

**Supplementary table 1.** Sequences of siRNA

| **Gene** | **Sense ( 5’-3’)** | **Antisense ( 5’-3’)** |
| --- | --- | --- |
| Si-PCK1#1 | CUCUGUCAAAGAUCGGCAUTT | AUGCCGAUCUUUGACAGAGTT |
| Si-PCK1#2 | GGAUGAAGUUUGACGCACATT | UGUGCGUCAAACUUCAUCCTT |
| Si-PCK1#3 | CAAAGAAUAAGCCAGAUGUTT | ACAUCUGGCUUAUUCUUUGTT |

**Supplementary table 2.** Sequences of qPCR primers

| **Gene** | **Forward Primer** | **Reverse Primer** |
| --- | --- | --- |
| PCK1 | GGAAGCCTGGACAGCCTACC | TCCTCAGAGCCGTCACAGATG |
| GAPDH | CATGTTCGTCATGGGGTGAACCA | AGTGATGGCATGGACTGTGGTCAT |
