# Supplementary material for: PCK1 as a potential hub gene in distinguishing lactate metabolism between rheumatoid arthritis and osteoarthritis
Source: PeerJ. 2025 Jul 31;13:e19661. doi: 10.7717/peerj.19661 (PMC12318502; doi:10.7717/peerj.19661)

Rabbit anti-human PCK1 Polyclonal antibody (Proteintech; catalog no. 16754-1-AP)
Observed molecular weight： 69 kDa

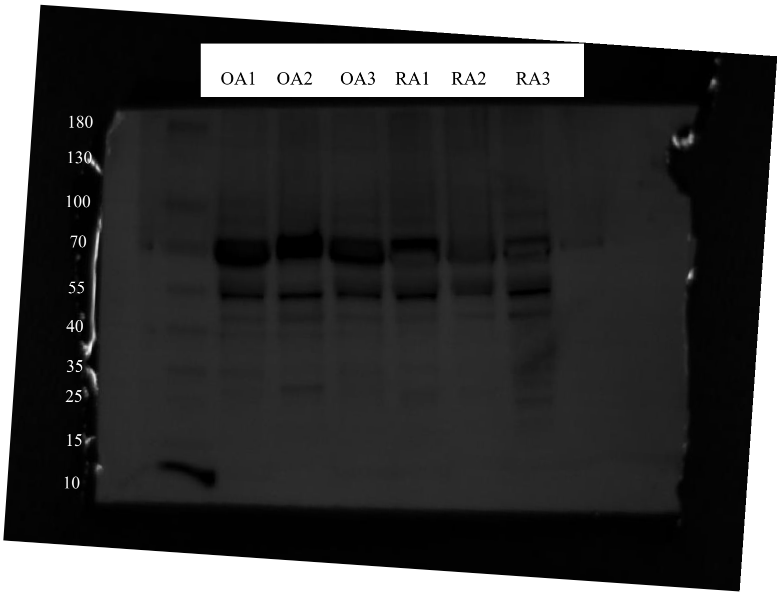

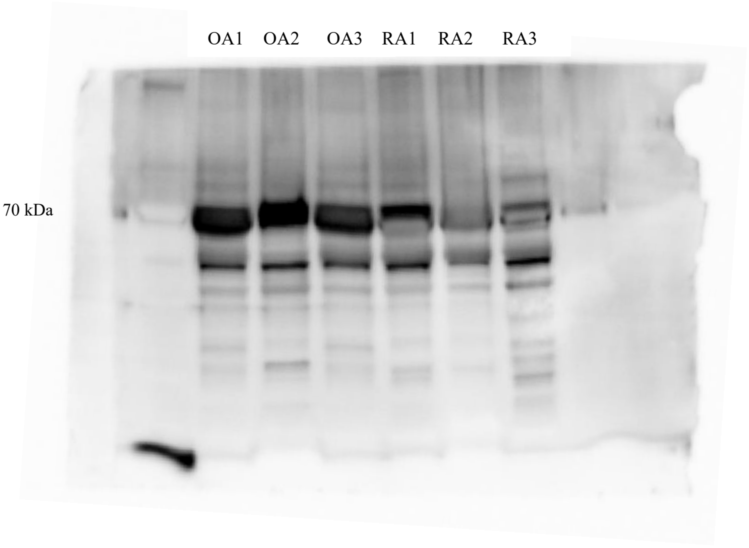

Mouse anti-human β-actin antibody (Proteintech; catalog no. 66009-1-Ig) Observed molecular weight： 42 kDa

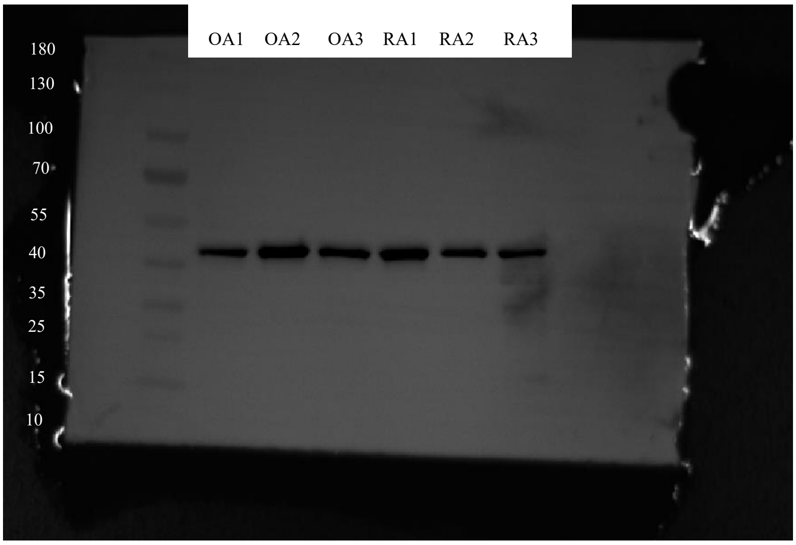

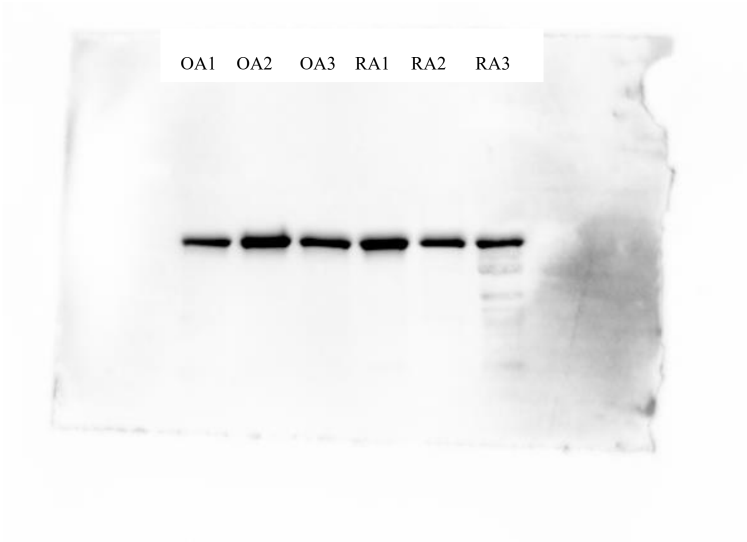

Supplement: Supplemental Information 6 [file peerj-13-19661-s006.pdf]
